# Light Intensity Modulates Locomotor Behavior and Predation in Different Color Morphs of the Harlequin Ladybird, *Harmonia axyridis*

**DOI:** 10.3390/insects16121280

**Published:** 2025-12-17

**Authors:** Xing-Xing Wang, Ya-Nan Liu, Chun-Yan Huang, Rui-Yan Li, Zhi-Wei Jiang, Chen-Yang Liu, Tong-Xian Liu, Yi Zhang

**Affiliations:** 1 Shandong Engineering Research Center for Environmentally Friendly Agricultural Pest Managemnent, College of Plant Health and Medicine, Qingdao Agricultural University, Qingdao 266000, China; wxx0521@qau.edu.cn (X.-X.W.); lyn2001@stu.qau.edu.cn (Y.-N.L.); 20220205470@stu.qau.edu.cn (C.-Y.H.); lry027@stu.qau.edu.cn (R.-Y.L.); 15562731283@163.com (Z.-W.J.); 13613128117@163.com (C.-Y.L.); 2 Institute of Entomology, Guizhou University, Guiyang 550025, China; tx.liu@gzu.edu.cn

**Keywords:** *Harmonia axyridis*, opsin, predatory behavior, color polymorphism

## Abstract

Light is a fundamental ecological cue for insects, influencing physiological rhythms and behavior. We investigated how varying light intensities affect locomotion and foraging in *H. axyridis* color morphs, and examined the role of visual opsins. Three adult female morphs were assayed under white light at 1000, 5000, and 10,000 lx. Higher light intensity significantly elevated body temperature and locomotor activity across morphs, with the inherently dark f. *conspicua* morph exhibiting the greatest increases. Predation rates on pea aphids trended upward with intensity but differed significantly by morph: f. *conspicua* beetles consistently consumed more prey than f. *succinea*. RNAi knockdown of the UV-sensitive opsin *HaUVSop-2* significantly reduced the crawling distance of satiated beetles under 5000 lux white light. Correspondingly, supplementation of white light with blue light (short wave) enhanced movement, whereas red supplementation increased aphid consumption. These results suggest that Short-wavelength light has the potential to stimulate the dispersal of ladybirds, whereas long-wavelength light may enhance predation on prey by increasing microenvironment temperature or improving prey recognition. We conclude that light intensity and spectrum jointly modulate *H. axyridis* behavior in a morph-dependent manner, mediated in part by visual opsins. Melanic morphs leverage thermal melanism to gain higher activity under bright light, implying an evolved trade-off between dispersal and stress tolerance. Our findings have practical implications: tailored lighting (e.g., blue-enhanced illumination to stimulate predation and dispersal of *H. axyridis*) could improve biological control efficacy in agroecosystems.

## Simple Summary

Light is a critical environmental factor for insects like the harlequin ladybird (*Harmonia axyridis*), a beneficial predator used in pest control. This study investigated how different light intensities and colors affect the behavior and predation efficiency of ladybirds with varying color patterns (melanic/dark vs. non-melanic/yellow morphs). We found that darker elytra ladybirds are generally more active and consume more aphids under all tested light intensities compared to yellow ladybirds, suggesting their high mobility is genetically determined, not just due to faster heating from the light. Increasing light intensity increased activity for all morphs. Furthermore, while silencing a specific ultraviolet-sensitive visual gene (*HaUVSop-2*) reduced movement in well-fed ladybirds, adding blue light increased their activity. These findings are valuable because they suggest that by adjusting the light environment in places like greenhouses, we can optimize the effectiveness of these natural enemy ladybirds for pest management.

## 1. Introduction

Among various physical stimuli, light serves as a critical environmental factor influencing insect physiology and behavior [1,2]. The effects of photoperiod and spectral composition have been extensively studied, leading to mature applications in pest control and the mass rearing of natural enemies. Photoperiod serves as a reliable proxy for seasonal progression, enabling insects to regulate voltinism, diapause, and migratory timing [3]. However, anthropogenic factors such as artificial light at night and skyglow can mimic long-day signals, causing seasonal misinterpretation and disrupting overwintering and reproductive rhythms [4]. In biological control programs, manipulating photoperiod based on these principles allows for the regulation of diapause to optimize storage, transport, and shelf life of arthropod natural enemies.

Spectral composition elicits distinct attraction or avoidance responses (phototaxis) across insect taxa. Ultraviolet (UV) and short-wave blue light are generally highly attractive to many nocturnal insects and pollinators, whereas green or yellow wavelengths often exert stronger effects on specific diurnal predators/parasitoids and herbivores. These behavioral responses form the biological basis for light trap devices widely applied in integrated pest management [5,6].

In contrast to photoperiod and spectrum, research focusing on light intensity is relatively scarce. Light intensity directly determines visibility and the temporal window for activity, thereby influencing foraging, dispersal, mating, and migration. Field and controlled experiments have demonstrated that high-intensity artificial light significantly alters the spatial distribution and survival of nocturnal insects; for instance, moth larval abundance and growth rates decline in proximity to streetlights [7]. High illuminance and broad-spectrum white light (e.g., LEDs) often exert stronger effects than traditional sodium vapor lamps, manifesting as increased attraction, disorientation, and mortality [7,8]. Physiologically, light intensity can modulate endocrine systems, affecting diapause induction, developmental rates, and reproductive maturation [4]. Mechanistically, light intensity operates through both visual photoreceptor activation and non-visual pathways (e.g., circadian clock genes), ultimately reshaping behavioral and physiological phenotypes.

Insects perceive and transduce photic signals through specialized visual systems, with opsins serving as the fundamental molecular basis. Opsins are G-protein-coupled receptors (GPCRs) with molecular weights typically between 30–50 kDa. They bind to a chromophore to form photopigments with specific spectral sensitivities [9,10,11,12]. Insect visual opsins are generally categorized into three clades based on peak sensitivity: UV-sensitive (UVSop, 300–400 nm), short-wave-sensitive (SWSop, 400–500 nm), and long-wave-sensitive (LWSop, 500–600 nm) [13]. Recent functional studies using RNAi and CRISPR have directly linked specific opsins to behaviors such as phototaxis and mate recognition [14].

The harlequin ladybird, *H. axyridis*, is a vital natural enemy in agricultural ecosystems. Its visual acuity is crucial for prey searching, mate recognition, and habitat selection [15]. The elytra of *H. axyridis* exhibit diverse color patterns, influenced by both genetics and environmental temperature [16]. Recent research indicates that *H. axyridis* exhibits significant phototactic responses to UV, blue, and green wavelengths, and opsin expression patterns may correlate with body coloration, affecting visual contrast against vegetative backgrounds [17,18]. Genomic analyses reveal that *H. axyridis* possesses two UV-sensitive opsin genes (*UVSop-1*, *UVSop-2*) and one long-wave-sensitive gene (*LWSop*), but lacks the blue-sensitive *SWSop* gene common in other orders [19]. A recent study by Qiu et al. [20] identified that *UVSop-2* (referred to as *UV2*) and *LWSop* plays key roles in wavelength-specific phototaxis in adults.

Despite progress in linking *H. axyridis* vision to molecular mechanisms, systematic research integrating color polymorphism, ecological niche, and light intensity is lacking. However, the interplay between light intensity and the feeding or locomotor activity of different genetic color morphs remains unclear. To address this, we investigated the effects of light intensity on the behavior of *H. axyridis* under controlled laboratory conditions. We compared locomotor parameters (crawling distance) and predation rates of adults and larvae with different elytral patterns (morphs) under varying light intensities and spectral compositions. Furthermore, we employed RNA interference (RNAi) to silence three opsin genes, verifying their specific roles in modulating behavior under photic stimulation.

## 2. Materials and Methods

### 2.1. Insects and Rearing Conditions

*H. axyridis* adults were collected from landscape trees in Qingdao, China (36°19′12″ N, 120°23′24″ E). The population was maintained in climate chambers at 25 °C, 65 ± 5% relative humidity (RH), and a 16L:8D photoperiod, fed with pea aphids (*Acyrthosiphon pisum*).

Induction of phenotypic plasticity in *H. axyridis* f. *succinea*:

To obtain f. *succinea* (yellow background) morphs with distinct melanization levels, eggs were reared under two thermal conditions. One group was reared at 15 °C to induce melanization (resulting in females with ~60% elytral melanization, referred to as “melanized f. *succinea*”), while another group was reared at 30 °C to produce non-melanized forms (females with no elytral spots, referred to as “non-melanized f. *sucinea*”). The melanic morph f. *conspicua* (black background with two red spots) was selected from the general population [21,22].

Prey:

Red morph pea aphids (*A. pisum*) were reared on broad bean plants (*Vicia faba*) under the same environmental conditions (25 °C, 16L:8D). Fourth-instar apterous aphids (N = 60) were used in predation assays to prevent aphid reproduction from confounding consumption counts in Petri dish (10 cm diameter), and leaf of *V. faba* was added for aphids rearing.

### 2.2. Behavioral and Predation Assays Under Varying Light Intensities

#### 2.2.1. Body Surface Temperature Measurement

**Figure 1:**
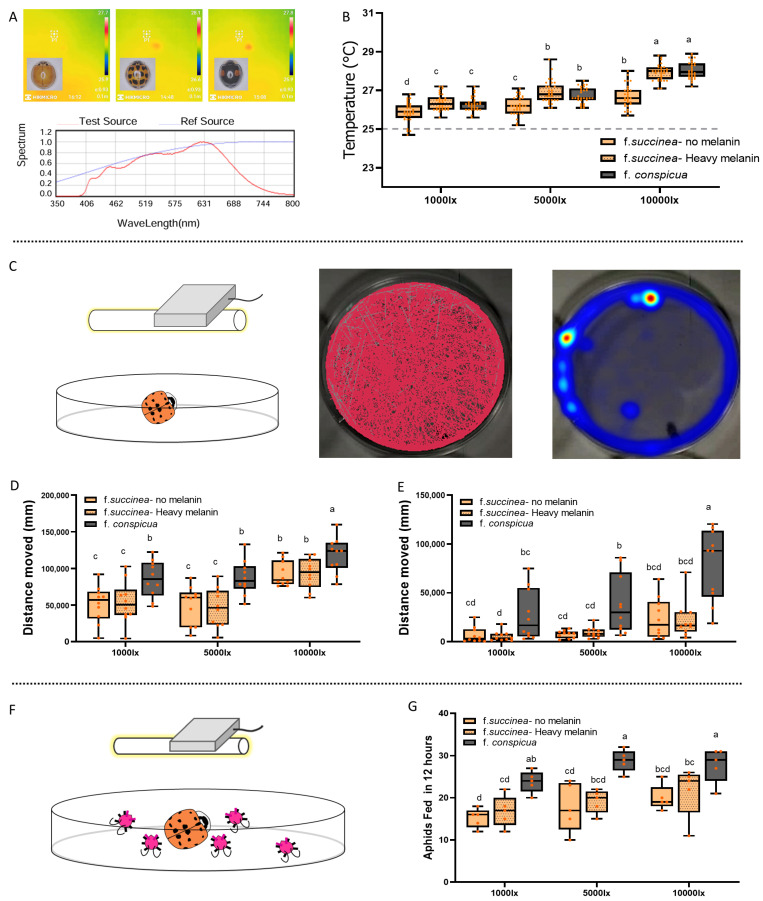
Effects of light intensity (1000, 5000, and 10,000 lx) on body temperature, locomotor activity, and predation capacity in three color morphs of *H. axyridis*. (**A**) The three selected *H. axyridis* adult morphs (top) and the spectral composition of the artificial light source (bottom). (**B**) Differences in body surface temperature under varying light intensities. (**C**) shows schematic of the locomotor activity assay and representative software tracking results (trajectory by red tracking line and heatmap by duration time). (**D**) Total distance moved over 3 h by starved individuals. (**E**) Total distance moved over 3 h by satiated individuals. (**F**) Schematic of the predation assay with aphids. (**G**) Number of aphids consumed over 12 h by starved adults. In the spectral curves shown in C, the red curve represents the spectral distribution of the measured target light source; the blue curve corresponds to the system’s built-in internal reference spectral curve. In panels (**B**,**D**,**E**,**G**), different letters indicate significant differences determined by ANOVA followed Tukey’s HSD post hoc test (*p* < 0.05).

Adult females (72 h post-emergence, unmated) of three morphs (non-melanized f. *succinea*, melanized f. *succinea*, and f. *conspicua*) were tested. The experimental arena was a side-vented Petri dish (10 cm diameter) lined with white filter paper. A variable LED light source (Lipro T20Q1, 4000 K, Ra97, MEIZU, Zhuhai, China; spectrum in Figure 1A) was adjusted to provide illuminance levels of 1000, 5000, and 10,000 lx, measured by a photometer (PLA-30, EVERFINE, Hangzhou, China). After 10 min of exposure at an ambient temperature of 25 °C, body surface temperature was recorded using a thermal imaging system (H21ProS+, HIKMICRO, Hangzhou, China). Emissivity was set to 0.93, with central point temperature measurement, macro mode, testing distance of 0.1 m, and rainbow color palette. Thirty individuals per morph were tested (n = 30).

#### 2.2.2. Locomotor Activity Analysis

Activity was assessed under the three light intensities (1000, 5000, 10,000 lx) using the same experimental setup. Behavior was recorded using a high-speed camera (MV-CS060, HIKROBOT, Hangzhou, China). The video recording lasted for 3 h.

Starved State: Beetles were starved for 24 h prior to testing.

Satiated State: Beetles were fed ad libitum for 3 h immediately prior to testing.

For each treatment, 10 biological replicates were recorded. Video files were analyzed using EthoVision XT (v16.0, Noldus, Wageningen, The Netherlands) to calculate total distance moved (cm) during the observation period.

#### 2.2.3. Predation Capacity Assay

Female adults (72 h post-emergence, starved for 24 h) of the three morphs were introduced into Petri dishes containing 50 fourth-instar aphids. The assay was conducted under 1000, 5000, and 10,000 lx at 25 °C. The number of consumed aphids was recorded after 12 h. Each treatment consisted of 5 replicates.

### 2.3. RNA Interference (RNAi) of Opsin Genes

#### 2.3.1. dsRNA Synthesis and Delivery

Double-stranded RNA (dsRNA) targeting *HaUVSop-1*, *HaUVSop-2*, and *HaLWSop* was synthesized using the T7 RiboMAX system (Promega, Madison, WI, USA). Primers containing T7 promoter sequences are listed in Table S1. A non-target control dsRNA was synthesized from a *Mus musculus* gene fragment (dsMus). One-week-old adults (f. *conspicua* females) received a microinjection of 250 nL dsRNA (~5000 ng/μL).

#### 2.3.2. Verification of RNAi Efficiency

Total RNA was extracted from beetle heads 48 h post-injection using RNAiso Plus (Takara, Tokyo, Japan). cDNA was synthesized using the PrimeScript™ RT reagent kit (Takara). Gene expression was quantified via RT-qPCR (LightCycler 96, Roche, Basel, Switzerland) using *HaRps3* as the reference gene. Relative expression was calculated using the 2^−ΔΔCt^ method (n = 3 replicates of 5 heads each).

#### 2.3.3. Behavioral Assays Post-RNAi

To assess the effect of knockdown of opsin gene on behavior, dsRNA was injected at the pupal stage (48 h post-pupation) and again 24 h post-emergence. Beetles were tested under 5000 lx white light. Locomotor activity was recorded for starved (24 h starvation) and satiated (fed for 6 h) individuals (n = 10). Predation was assessed over 12 h using starved individuals (n = 5).

### 2.4. Effect of Spectral Composition Supplementation

Using f. *conspicua* females (72 h post-emergence, unmated), narrowband monochromatic light was added to a basal white light (1000 lx) to enhance specific wavelengths. Building on the findings reported in Qiu et al.’s study, which demonstrated that the visual proteins of *H. axyridis* primarily exhibit strong behavioral responses to visible light (with *HaUVSop-2* specifically responsive to the blue light spectrum) [20], three distinct wavelength bands within the visible spectrum were selected for subsequent treatments. The supplemental lights were Blue (473 nm), Green (519 nm), and Red (631 nm), each adjusted to add about 600 lx. Locomotor activity and predation were measured as described in Section 2.2.2 and Section 2.2.3 (n = 10 for locomotor activity, n = 5 for predation).

### 2.5. Statistical Analysis

Data were analyzed using SPSS (v26.0). Normality and homogeneity of variance were checked. Research data with two factors were analyzed using two-way ANOVA. Differences between groups were analyzed using one-way ANOVA followed by Tukey’s HSD post hoc test, or Student’s *t*-test for pairwise comparisons. Data are presented as mean ± standard error (SE).

## 3. Results

### 3.1. Behavior and Predation of H. axyridis Color Morphs Under Varying Light Intensities

#### 3.1.1. Body Surface Temperature

Body surface temperatures differed significantly among color morphs and light intensities. While increased light intensity raised body temperature across all morphs, the melanic forms (f. *conspicua* and melanized f. *succinea*) exhibited significantly higher temperatures than the non-melanized f. *succinea* at all tested intensities. A two-way ANOVA was conducted to examine the effect of light intensity and ladybird forms on body temperatures, there was a significant interaction between these two factors (F = 9.284, df = 4, 261, *p* < 0.001; Figure 1B). Both light intensity and color morph significantly affected body temperature, and thermal plasticity induced by rearing temperature in f. *succinea* resulted in thermal properties similar to the genetically melanic f. *conspicua* (F = 198.447, df = 2, 261, *p* < 0.001 and F = 85.022, df = 2, 261, *p* < 0.001; Figure 1B).

#### 3.1.2. Locomotor Activity

Locomotor activity was positively correlated with light intensity. In the starved state, crawling distance increased with intensity, A two-way ANOVA was conducted to examine the effect of light intensity and ladybird forms on crawling distances. There was no significant interaction between these two factors (F = 0.291, df = 4, 81, *p* = 0.883; Figure 1D). The results indicated significant main effects of both light intensity and color morph on locomotor activity (F = 25.369, df = 2, 81, *p* < 0.001 and F = 17.721, df = 2, 81, *p* < 0.001; Figure 1D).

In the satiated state, crawling distance also increased with intensity, however, there was a significant interaction between light intensity and ladybird forms (F = 2.593, df = 4, 81, *p* = 0.043; Figure 1E). Results showed that for both light intensity and ladybird forms had a significant effect (F = 15.855, df = 2, 81, *p* < 0.001 and F = 29.289, df = 2, 81, *p* < 0.001; Figure 1E).

#### 3.1.3. Predation Capacity

A two-way ANOVA was conducted to examine the effect of light intensity and ladybird forms on predation rates over 12 h. There was no significant interaction between these two factors (F = 4.956, df = 4, 36, *p* = 0.859; Figure 1G). Furthermore, both light intensity and color morph significantly influenced predation rates. Specifically, f. *conspicua* consumed significantly more aphids than f. *succinea* across all light treatments (F = 5.429, df = 2, 36, *p* = 0.009 and F = 23.538, df = 2, 36, *p* < 0.001; Figure 1G).

### 3.2. Role of Opsin Genes in Behavior and Predation

#### 3.2.1. RNAi Efficiency and Behavioral Changes After Knockdown of Opsin Gene

**Figure 2:**
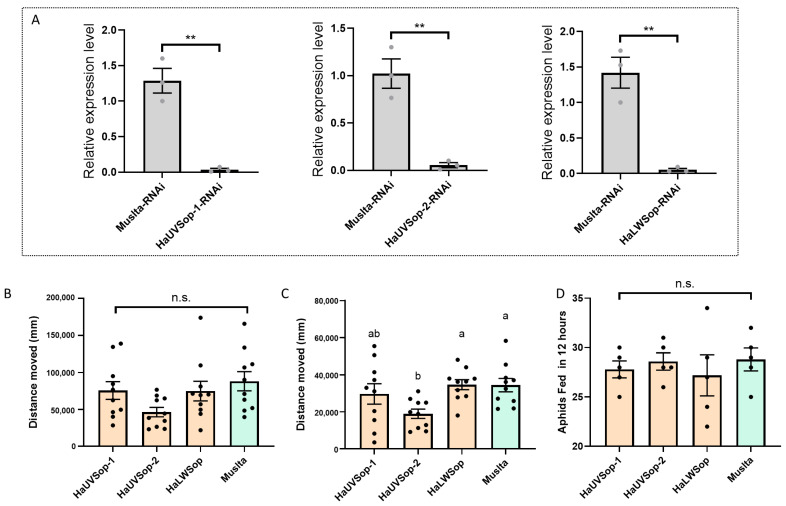
Effects of RNA interference (RNAi) targeting three visual opsin genes on gene expression, behavior, and predation in *H. axyridis* (only f. *conspicua* females were used in this study). (**A**) RNAi efficiency showing relative expression levels of *HaUVSop-1*, *HaUVSop-2*, and *HaLWSop*. Asterisks (**) indicate highly significant differences compared to controls (*t*-test, *p* < 0.001). (**B**) Locomotor activity (distance moved) of starved beetles following knockdown of opsin gene. (**C**) Locomotor activity of satiated beetles following knockdown of opsin gene. (**D**) Predation capacity (aphids consumed in 12 h) following knockdown of opsin gene. Different letters in panel C indicate significant differences determined by one-way ANOVA (*p* < 0.05); n.s. indicates no significant difference.

dsRNA injection successfully downregulated target transcripts compared to the ds-Muslta control: *HaUVSop-1* by about 98% (t = 7.168, df = 4, *p* = 0.002; Figure 2A), *HaUVSop-2* by about 95% (t = 6.157, df = 4, *p* = 0.0035; Figure 2A), and *HaLWSop* by about 96% (t = 6.258, df = 4, *p* = 0.0033; Figure 2A).

#### 3.2.2. Behavioral Changes After Knockdown of Opsin Genes

Under 5000 lx white light, silencing *HaUVSop-1* or *HaLWSop* did not significantly alter crawling distance in either starved or satiated states compared to controls (F = 2.356, df = 3, 36, *p* = 0.0878; Figure 2B). In contrast, silencing *HaUVSop-2* significantly reduced the locomotor activity of satiated beetles, while having no significant effect on starved individuals (F = 3.794, df = 3, 36, *p* = 0.0184; Figure 2C).

Regarding predation, no significant differences in aphid consumption were observed following the knockdown of any of the three opsin genes (F = 0.3045, df = 3, 16, *p* = 0.8217; Figure 2D).

### 3.3. Effect of Spectral Supplementation

**Figure 3:**
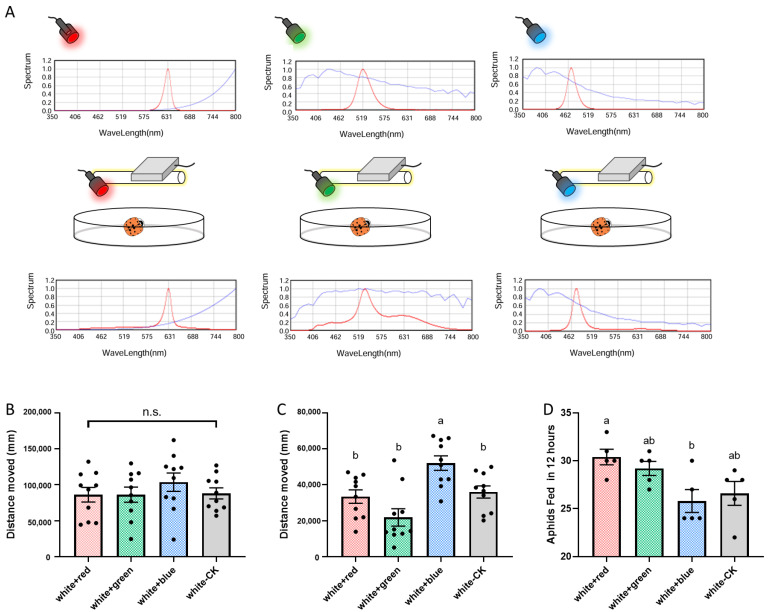
Effects of supplementing white light with monochromatic narrowband light (Red: 631 nm, Green: 519 nm, Blue: 473 nm) on *H. axyridis* behavior and predation. (**A**) Spectral composition of the white light source supplemented with the respective monochromatic lights. (**B**) Locomotor activity (distance moved) of starved beetles. (**C**) Locomotor activity of satiated beetles. (**D**) Predation capacity (aphids consumed in 12 h). Different letters in (**C**,**D**) indicate significant differences determined by one-way ANOVA (*p* < 0.05); n.s. indicates no significant difference. In the spectral curves shown in A, the red curve represents the spectral distribution of the measured target light source; the blue curve corresponds to the system’s built-in internal reference spectral curve. For red light detection, the internal reference curve is the blackbody radiation curve; for green light detection, it is the CIE Standard Daylight Model; and for blue light detection, the internal reference curve is based on an extended theoretical daylight model.

Supplementing white light with specific wavelengths elicited distinct behavioral responses. Adding Blue light (473 nm) significantly increased the crawling distance of satiated beetles (starved: F = 0.6529, df = 3, 36, *p* = 0.5863; satiated: F = 9.511, df = 3, 36, *p* < 0.001; Figure 3B,C), mirroring the effect observed in the *HaUVSop-2* RNAi experiment. Green and Red supplementation did not alter locomotor activity. In the treatment groups with red light adding, ladybirds exhibited higher feeding rates compared to those exposed to blue light adding, and exhibits significant difference (F = 4.444, df = 3, 16, *p* = 0.0188; Figure 3D).

## 4. Discussion

Our results demonstrate that light intensity is a key regulator of *H. axyridis* behavior, with strong interactions between morph coloration and response. This study supports the hypothesis of a trade-off strategy within the species [23]. As expected, increased light intensity raised body temperatures, with dark morphs heating faster than light morphs, consistent with the thermal melanism hypothesis [24,25]. However, thermal effects alone do not explain behavioral differences. Under identical light intensities, the environmentally induced melanized f. *succinea* and the genetically melanic f. *conspicua* reached similar body temperatures, yet only f. *conspicua* showed hyperactivity. Thus, f. *conspicua*’s high mobility appears to be a genetically determined trait, not merely a thermodynamic consequence. This suggests a trade-off strategy within the species [23]: the yellow f. *succinea* form may be adapted for stress resistance (e.g., cold tolerance and winter survival), while the melanic f. *conspicua* invests in rapid dispersal and intense foraging during favorable conditions. Such polymorphic strategies likely enhance *H. axyridis*’ invasiveness by allowing exploitation of diverse niches [17].

It should be noted that the varying degrees of melanization in the f. *succinea* population in this study were achieved through temperature stimulation at different developmental stages. Therefore, developmental temperature may affect biological characteristics beyond elytral coloration. However, compared to the f. *conspicua* population, the f. *succinea* populations under different environmental conditions showed no significant differences in locomotor behavior activity or feeding capacity, which indirectly suggests the important role of genetic background in these traits.

Previous work by Qiu et al. [20] established that *HaUVSop-2* and *HaLWSop* are the primary drivers of wavelength-specific phototaxis (directional movement), finding no significant role for *HaUVSop-1* in spectral preference. Our study reveals for *HaUVSop-2*: modulating general locomotor activity (kinesis) in response to light intensity, particularly in satiated individuals. Silencing *HaUVSop-2* reduced movement in satiated beetles, and blue light supplementation enhanced it. This suggests that *HaUVSop-2* may function as a directional guide based on phototaxis or a dispersal trigger. Under starvation, the drive to forage likely overrides light-mediated dispersal signals, which may explain why opsin gene knockdown did not significantly affect locomotor activity in starved ladybirds.

While opsin silencing did not significantly impair predation in a small arena—likely due to the dominance of olfactory and gustatory cues at close range—supplementing with red light significantly improved predation efficiency. However, it should also be noted that regarding the effects induced by red light, which is generally undetectable for *H. axyridis* [20], we hypothesize that as the selected light source’s spectral range could be partially detected by the corresponding long-wavelength visual proteins, and it may be attributed to enhanced contrast between aphids and their environment [18]. Secondly, as mentioned in our discussion, the limited spatial scale of the experimental setup increased the probability of random encounters with aphids, while chemical perception (e.g., gustatory and olfactory cues) likely played a more dominant role [26]. Additionally, given red light’s stronger thermal effect, prolonged exposure may have elevated microenvironmental temperature, indirectly increasing ladybird activity levels and consequently enhancing feeding rates.

These findings have practical implications for biological control. In protected agriculture (e.g., greenhouses), manipulating the light environment could optimize biocontrol efficacy. For ladybirds *H. axyridis,* increasing blue-rich lighting may stimulate dispersal and searching behavior in released. This approach could be extended to other natural enemies once their behavioral responses to photic stimulation are characterized. Furthermore, recognizing the higher efficacy of melanic morphs of *H. axyridis* under high-light conditions suggests that strain selection could be tailored to specific environmental release conditions [27].

## 5. Conclusions

In summary, *H. axyridis* with different genetic backgrounds exhibit significant differences in activity and predatory ability. Short-wavelength light signals serve as key external regulatory factors, and the opsin proteins responsible for short-wavelength perception play a crucial role in signal transduction. Theoretically, this study reveals an ecological “trade-off” adaptation strategy within the species [23]. From an applied perspective, light intensity and spectrum jointly determine both the immediate behavioral effects and long-term population consequences of artificial light on insects. In agricultural and urban management contexts, the development of lighting control and harm mitigation designs based on spectral/shielding strategies has demonstrated potential [4,28]. Optimizing light management strategies for natural enemy insects, combined with selective releasing, holds potential for regulating their dispersal capacity and predation efficiency [8,28].

## Data Availability

The original contributions presented in this study are included in the article/Supplementary Materials. Further inquiries can be directed to the corresponding author.

## Supplementary Materials

The following supporting information can be downloaded at: https://www.mdpi.com/article/10.3390/insects16121280/s1, Table S1: List of primers utilized in this study.
