# Supplementary material for: Light Intensity Modulates Locomotor Behavior and Predation in Different Color Morphs of the Harlequin Ladybird, Harmonia axyridis
Source: Insects. 2025 Dec 17;16(12):1280. doi: 10.3390/insects16121280 (PMC12733748; doi:10.3390/insects16121280)
Supplement: Supplementary file 1 [file insects-16-01280-s001.zip › insects-4026798-supplementary.pdf]

*Article*

# Light Intensity Modulates Locomotor Behavior and Predation in Different Color Morphs of the Harlequin Ladybird, *Harmonia axyridis*

**Xing-Xing Wang [1], Ya-Nan Liu [1], Chun-Yan Huang [1], Rui-Yan Li [1], Zhi-Wei Jiang [1], Chen-Yang Liu [1], Tong-Xian Liu [2] and Yi Zhang [1,*]**

1 Shandong Engineering Research Center for Environmentally Friendly Agricultural Pest Management, College of Plant Health and Medicine, Qingdao Agricultural University, Qingdao 266000, China; wxx0521@qau.edu.cn (X.-X.W.); lyn2001@stu.qau.edu.cn (Y.-N.L.); 20220205470@stu.qau.edu.cn (C.-Y.H.); lry027@stu.qau.edu.cn (R.-Y.L.); 15562731283@163.com (Z.-W.J.); 13613128117@163.com (C.-Y.L.)

2 Institute of Entomology, Guizhou University, Guiyang 550025, China; tx.liu@gzu.edu.cn

* Correspondence: yizhang085@qau.edu.cn

**Table S1.** List of primers utilized in this study.

| | Forward (5' to 3') | Reverse (5' to 3') | PCR |
|---|---|---|---|
| Q-*UVSop-1* | GCCAGCTGTATAGATCCCTT | AGTTTGAATTGGCATCATCG | RT-qPCR |
| Q-*UVSop-2* | TCAAAGAAGTTTGGACATGC | CCATCCGAGTCTATGAAGAA | RT-qPCR |
| Q-*HaLWSop* | TAGGTATAGGAATTCTGGGCTT | AAATGGATTCCCAAAGATGGA | RT-qPCR |
| Q-*HaRps3* (Ref) | GGCTACCAGAACCGAACAGAG | GTGCTATGGCGCATAATCCT | RT-qPCR |
| ds-*UVSop-1* | T7+GGCTCCCAAAGAGAGAAACGA | T7+GCCAACCTCCTTATGCTGGA | dsRNA |
| ds-*UVSop-2* | T7+AATGGGTTCGGGGGAAAATGA | T7+GTTGGAAGCTGTTCTCAGGC | dsRNA |
| ds-*HaLWSop* | T7+GTGTGCAGTGACTTTTCCGT | T7+GGGGGTACTGTTAGAGCCAA | dsRNA |
| ds-*Muslta* | T7+CACCCTCTCCACGAATTG | T7+TAGAAGATGCTGCTGTTTCA | dsRNA |
